# Neutrophil extracellular traps contribute to the pathogenesis of leprosy type 2 reactions

**DOI:** 10.1371/journal.pntd.0007368

**Published:** 2019-09-10

**Authors:** Camila Oliveira da Silva, André Alves Dias, José Augusto da Costa Nery, Alice de Miranda Machado, Helen Ferreira, Thais Fernanda Rodrigues, João Pedro Sousa Santos, Natalia Rocha Nadaes, Euzenir Nunes Sarno, Elvira Maria Saraiva, Verônica Schmitz, Maria Cristina Vidal Pessolani

**Affiliations:** 1 Laboratório de Microbiologia Celular, Instituto Oswaldo Cruz, Fundação Oswaldo Cruz (FIOCRUZ), Rio de Janeiro, Rio de Janeiro, Brazil; 2 Laboratório de Hanseníase, Instituto Oswaldo Cruz, Fundação Oswaldo Cruz (FIOCRUZ), Rio de Janeiro, Rio de Janeiro, Brazil; 3 Laboratório de Imunobiologia das Leishmanioses, Departamento de Imunologia, Instituto de Microbiologia Paulo de Góes, Universidade Federal do Rio de Janeiro (UFRJ), Rio de Janeiro, Rio de Janeiro, Brazil; University of Liverpool, UNITED KINGDOM

## Abstract

Up to 50% of patients with the multibacillary form of leprosy are expected to develop acute systemic inflammatory episodes known as type 2 reactions (T2R), thus aggravating their clinical status. Thalidomide rapidly improves T2R symptoms. But, due to its restricted use worldwide, novel alternative therapies are urgently needed. The T2R triggering mechanisms and immune-inflammatory pathways involved in its pathology remain ill defined. In a recent report, we defined the recognition of nucleic acids by TLR9 as a major innate immunity pathway that is activated during T2R. DNA recognition has been described as a major inflammatory pathway in several autoimmune diseases, and neutrophil DNA extracellular traps (NETs) have been shown to be a prime source of endogenous DNA. Considering that neutrophil abundance is a marked characteristic of T2R lesions, the objective of this study was to investigate NETs production in T2R patients based on the hypothesis that the excessive NETs formation would play a major role in T2R pathogenesis. Abundant NETs were found in T2R skin lesions, and increased spontaneous NETs formation was observed in T2R peripheral neutrophils. Both the *M*. *leprae* whole-cell sonicate and the CpG-Hlp complex, mimicking a mycobacterial TLR9 ligand, were able to induce NETs production *in vitro*. Moreover, TLR9 expression was shown to be higher in T2R neutrophils, suggesting that DNA recognition via TLR9 may be one of the pathways triggering this process during T2R. Finally, treatment of T2R patients with thalidomide for 7 consecutive days resulted in a decrease in all of the evaluated *in vivo* and *ex vivo* NETosis parameters. Altogether, our findings shed light on the pathogenesis of T2R, which, it is hoped, will contribute to the emergence of novel alternative therapies and the identification of prognostic reactional markers in the near future.

## Author summary

Leprosy is caused by a mycobacterium that has a predilection for skin and nerve cells. The chronic course of the disease may be interrupted by acute inflammatory episodes known as reactions, despite effective bacterial killing with antibiotics. Reactions aggravate the patient’s clinical status and may become a medical emergency. Type 2 reactions (T2R) only occur in patients with high bacterial burden and are treated with thalidomide and/or corticosteroids. We are interested in understanding how inflammation is triggered and amplified during T2R. In this study we investigated the potential role of extracellular DNA released by neutrophils (known as NETs) in T2R, since they have been shown to cause inflammation. Abundant NETs were found in T2R skin lesions, and increased spontaneous NETs formation was observed in neutrophils present in the blood of T2R patients. Moreover, bacterial constituents were able to induce NETs production. Finally, treatment of T2R patients with thalidomide resulted in decreased NET formation. Altogether, our findings shed light on the pathogenesis of T2R, which, it is hoped, will contribute to the identification of biomarkers for early diagnosis and emergence of novel alternative therapies in the near future.

## Introduction

Leprosy, a disease widely associated with debilitating disfiguration, remains a public health threat in several low- and middle-income countries, including Brazil, with approximately 27,000 new cases each year [1]. The disease is caused by the obligate intracellular pathogen *Mycobacterium leprae* (ML) that targets skin macrophages and Schwann cells in the peripheral nerves as its preferential niche. Leprosy manifests as a spectrum of clinical forms in consonance with the immune response built by the host against the infection. In the multibacillary (MB) forms of the disease (lepromatous [LL], borderline lepromatous [BL]), patients generate weak specific cellular immunity, resulting in uncontrolled ML proliferation, numerous lesions, and extensive skin and nerve infiltration [2]. The chronic course of MB leprosy may be interrupted by acute inflammatory episodes known as type 2 reactions (T2R), the primary causes of peripheral nerve damage. T2R affects 30–50% of MB patients, and erythema nodosum leprosum (ENL) presents as the most frequent manifestation of T2R. Characterized by painful cutaneous nodules or lesions, fever, joint and bone pain, iridocyclitis, neuritis, dactylitis, lymphadenitis, orchitis, and nephritis, and, in several aspects, often resembling typical chronic autoimmune diseases such as systemic lupus erythematosus (SLE) and rheumatoid arthritis, T2R presents as a systemic inflammation whose clinical symptoms range from mild to severe [3–5].

LL leprosy and a bacterial index (BI) of ≥ 4 are risk factors for developing T2R. Patients may present with T2R as their first manifestation of leprosy, but it is most frequently observed after the initiation of anti-mycobacterial multidrug therapy (MDT). T2R often runs a recurrent or chronic course, sometimes for many years, despite successful completion of MDT. In many countries, steroids are used to treat T2R. High doses are often required for long periods of time even though they do not always control the inflammation. In contrast, because treatment with thalidomide rapidly improves the major T2R symptoms, it is considered the drug of choice. But, due to its teratogenic effects, Brazil is one of the few countries in which it is allowed for leprosy treatment [4,6].

Nonetheless, to this day, the T2R triggering mechanisms and immune-inflammatory pathways involved in its pathology remain ill defined. T2R is characterized by elevated levels of pro-inflammatory cytokines both at the lesion sites and systemically. The possible roles played by immune complex deposition and complement system activation in addition to that of specific cell-mediated immunity have been described [6,7]. Based on current evidence, it is reasonable to hypothesize that neutrophils play a central role in T2R pathogenesis. In contrast to non-reactional MB patients, T2R patients classically show an intense perivascular neutrophil infiltrate throughout the dermis and subcutis accompanied by neutrophilia [8]. Likewise, T2R peripheral neutrophils have been shown to display an activated phenotype in light of higher CD64 level expression [9] and the release of TNF [10,11]. However, the precise mechanisms by which these cells contribute to the uncontrolled inflammation observed in T2R episodes remain nebulous.

In a recent report, we defined the recognition of nucleic acids by TLR9 as a major innate immunity pathway that is activated during T2R. Both ML and endogenous DNA-histone complexes were found at higher levels in the serum of T2R patients [12]. DNA recognition has been described as a major inflammatory pathway in several autoimmune diseases [13]. Particularly in SLE, it has been shown that neutrophil DNA extracellular traps (NETs), composed by a DNA scaffold decorated with proteins from different neutrophil compartments, are a prime source of endogenous DNA [14]. Considering that neutrophil abundance is a marked characteristic of T2R lesions, the objective of this study was to investigate NETs production in T2R patients based on the hypothesis that the excessive NETs formation will trigger the activation and amplification of the immune-inflammatory pathways enrolled in its physio-pathogenesis.

## Methods

### Ethics statement

This study was approved by the Ethics Committee of FIOCRUZ (CAAE 56113716.5.0000.5248). Informed written consent was obtained from all patients and healthy volunteers prior to specimen collection. All recruited individuals in this study were adults.

### Patient recruitment

Leprosy patients were recruited at the Souza Araújo Outpatient Unit (Reference Center for Leprosy Diagnosis and Treatment, Laboratório de Hanseníase, Fundação Oswaldo Cruz, Rio de Janeiro, RJ, Brazil) from October 2016 thru December 2018. The patients were classified on the leprosy spectrum clinically and histologically based on Ridley-Jopling classification schemes [15] and were administered WHO-recommended leprosy multidrug treatment (MDT). The study population consisted of 2 groups of patients: i) LL/BL patients recruited before the start of MDT with no signs of reaction at the time of leprosy diagnosis (n = 17: 11 LL and 6 BL, 10 men, 7 women, aged 22–80 with a median age of 50); and ii) T2R patients (n = 23: 22 LL and 1 BL, 17 men, 6 women, aged 24–76 with a median age of 40) recruited at diagnosis of reaction. T2R patients were clinically diagnosed as ENL (n = 19) or erythema multiforme (EM; n = 4) according to the type of skin lesions present at the dermatological examination. The so called “iris” or target lesion was considered a primary characteristic of EM, while dermal erythematous nodules a characteristic of ENL [3,16]. Reaction occurred before (n = 3), during (n = 9), or after ending MDT (n = 11). EM and ENL patients were treated with thalidomide at 100–300 mg/day and 16 were recruited for a reevaluation at day 7 of treatment (T2R_thal_). Healthy donors (HD) were also included (6 men, 10 women, aged 21–64, with a median age of 27) and participated in different assays during the study. None of the T2R patients had been treated with corticosteroid and/or thalidomide for at least 4 months prior to recruitment.

### Neutrophil isolation

Human neutrophils were isolated under endotoxin-free conditions from heparinized venous blood after density gradient centrifugation by Ficoll-Paque (GE Healthcare Life Sciences, NJ, USA). The neutrophil-rich layer was collected and residual erythrocytes were removed by lysis with an ACK (Ammonium-Chloride-Potassium) buffer. The quality of the neutrophil preparations was assessed by light microscopy of cytocentrifuged preparations, as previously described [10], and by flow cytometry using a mAb against CD16 conjugated with PE-Cy7 (catalog number 25-0168-42/clone CB16; BD Bioscience, CA, USA). The cells were assessed via the FACS Accuri flow cytometer (BD Bioscience); and the resulting data were analyzed by way of FlowJo V10 software (Tree Star). Neutrophil viability was estimated by way of Trypan blue-dye exclusion.

### Quantification of NET-DNA in culture supernatants

Purified neutrophils (> 95% of the cells) were resuspended in RPMI medium 1640 (GIBCO Life technologies, MA, USA) and (1x10^6^) incubated at 37°C in centrifuge microtubes (Eppendorf, NY, USA) with or without *M*. *leprae* whole-cell sonicate (MLWCS; 20 μg/ml; NR-19329; Bei Resources, VA, USA), CpG-Hlp complex (rHlp: 0.25 μM; CpG 2395: 0.5 μM; Invivogen, CA, USA) or phorbol 12-myristate 13-acetate (PMA; Sigma-Aldrich). The preparation of the CpG-Hlp complex was performed as previously described [12]. In the case of *in vitro* treatment with thalidomide (Abcam, CB, UK), the drug was dissolved in DMSO (Sigma-Aldrich, MO, EUA) and used at a final concentration of 50 μg/ml. NETs were quantified in the culture supernatant via the Quant-iT PicoGreen dsDNA Assay kit (Thermo Fisher Scientific, MA, USA) according to the manufacturer’s instructions. *In vitro* thalidomide efficacy was tested in human monocytes stimulated with LPS (Sigma-Aldrich), as previously described [17]. Neutrophil supernatants were also tested for lactate dehydrogenase activity (LDH) by using the Liquiform LDH kit (LABTEST, MG, Brazil) according to the manufacturer’s instructions. NADH oxidation was monitored at 340 nm in the Eon High Performance Microplate Spectrophotometer (BIOTEK, VT, USA).

### NETs detection by immunofluorescence

Immunofluorescence staining in frozen skin tissue was performed, as previously described [12]. For *in vitro* immunofluorescence staining, neutrophils (2x10^6^) were seeded in 24-well plates containing poly-L-lysine-coated coverslips (1:10, Sigma-Aldrich), followed by stimulation with MLWCS, CpG-Hlp (variable concentrations) or 200 ng/mL PMA for 90 min and fixation with 4% paraformaldehyde. NETs were evidenced by DAPI (Sigma-Aldrich), rabbit polyclonal anti-myeloperoxidase (MPO) antibody (1:50 for *in vitro* immunofluorescence; 1:100 for tissue; catalog number sc-16128-R; Santa Cruz Biotechnology, CA, EUA), and mouse monoclonal anti-histone H1 antibody (1:100; catalog number 05-457/clone AE-4; Merck Millipore, MA, EUA). The tissue sections and *in vitro* neutrophils were then incubated with secondary antibodies: IgG anti-rabbit conjugated to Alexa Fluor 488 and IgG2a anti-mouse conjugated to Alexa Fluor 633 for neutrophils, and IgG anti-rabbit conjugated to Alexa Fluor 633 and IgG 2a anti-mouse conjugated to Alexa Fluor 594 for tissue (Molecular Probes, OR, USA). Secondary antibodies were used at the concentration of 1:1000. Coverslips were mounted with Permafluor (Thermo Scientific), sealed with mounting medium (Permount Mounting Medium; Fisher Chemical/ Fisher Scientific). Images were obtained via an AxiObserver Z1 Colibri microscope (Zeiss, NI, Germany) and processed by AxioVision software (Zeiss). The percentage of NETs-releasing cells was calculated as an average of 5 random fields normalized to the total number of neutrophils as previously described [18].

### TLR9 expression

Neutrophils (5x10^5^) were fixed with 4% paraformaldehyde and stored refrigerated (4°C) until use. The cells were centrifuged, suspended in PBS containing 1% fetal calf serum (GIBCO Life technologies) and an Fc receptor-blocking solution (1:20; human TruStainFcX; Biolegend, CA, USA). The neutrophils were then permeabilized with 0.1% saponin and incubated with a mAb against TLR9 conjugated with FITC (1:40; catalog number ab134369/clone 26C593.2; Abcam) or an isotype control and labeling assessed using FACS Accuri flow cytometer (BD Bioscience). The resulting data were analyzed by FlowJo V10 software (Tree Star).

### Quantification of serum DNA-histone and DNA-MPO complexes

The levels of human histone (H1, H2A, H2B, H3, and H4)-associated DNA fragments in the serum were quantified by a photometric enzyme immunoassay (Roche Life Science, IN, USA); and analyzed as determined by the manufacturer.

For the quantification of the DNA-MPO complex levels in the serum samples, a previously described ELISA was performed [19] The anti-MPO antibody used in our assays was a different one (catalog number sc-16128-R; Santa Cruz Biotechnology, TX, USA).

### Statistical analysis

Statistical analysis was performed with GraphPad Prism version 6 (GraphPad Software). Differences between 2 groups of not normally distributed data were examined by Mann Whitney test. The Wilcoxon test was used to compare paired samples. Comparisons between >2 groups of not normally distributed data were examined by way of the Kruskal-Wallis test with Dunn multiple comparison post test. Spearman correlation coefficient was used to assess association between DNA-histone and DNA-MPO complexes; TLR9 expression and DNA-histone or DNA-MPO complexes; and for association analysis of BI and DNA-histone or DNA-MPO complexes. The adopted statistical significance level was P<0.05.

## Results and discussion

The potential role of NETs in the pathogenesis of leprosy T2R was assessed in 23 T2R patients in comparison with 17 non-reactional LL/BL controls. Blood samples and biopsies of skin lesions were obtained. All patients recruited in this study were in attendance at the Outpatient Unit of the Oswaldo Cruz Foundation (Fiocruz), Rio de Janeiro, RJ, Brazil. LL/BL patients were recruited upon leprosy diagnosis and before initiating MDT. T2R patients were enrolled in the study at diagnosis of reaction and initiation of thalidomide treatment. Fifteen T2R patients were reassessed at day 7 of treatment (T2R_thal_) when a clear remission of T2R symptoms was observed in all of them. Healthy donors (HD) participated in a variety of assays during the study.

**Fig 1:**
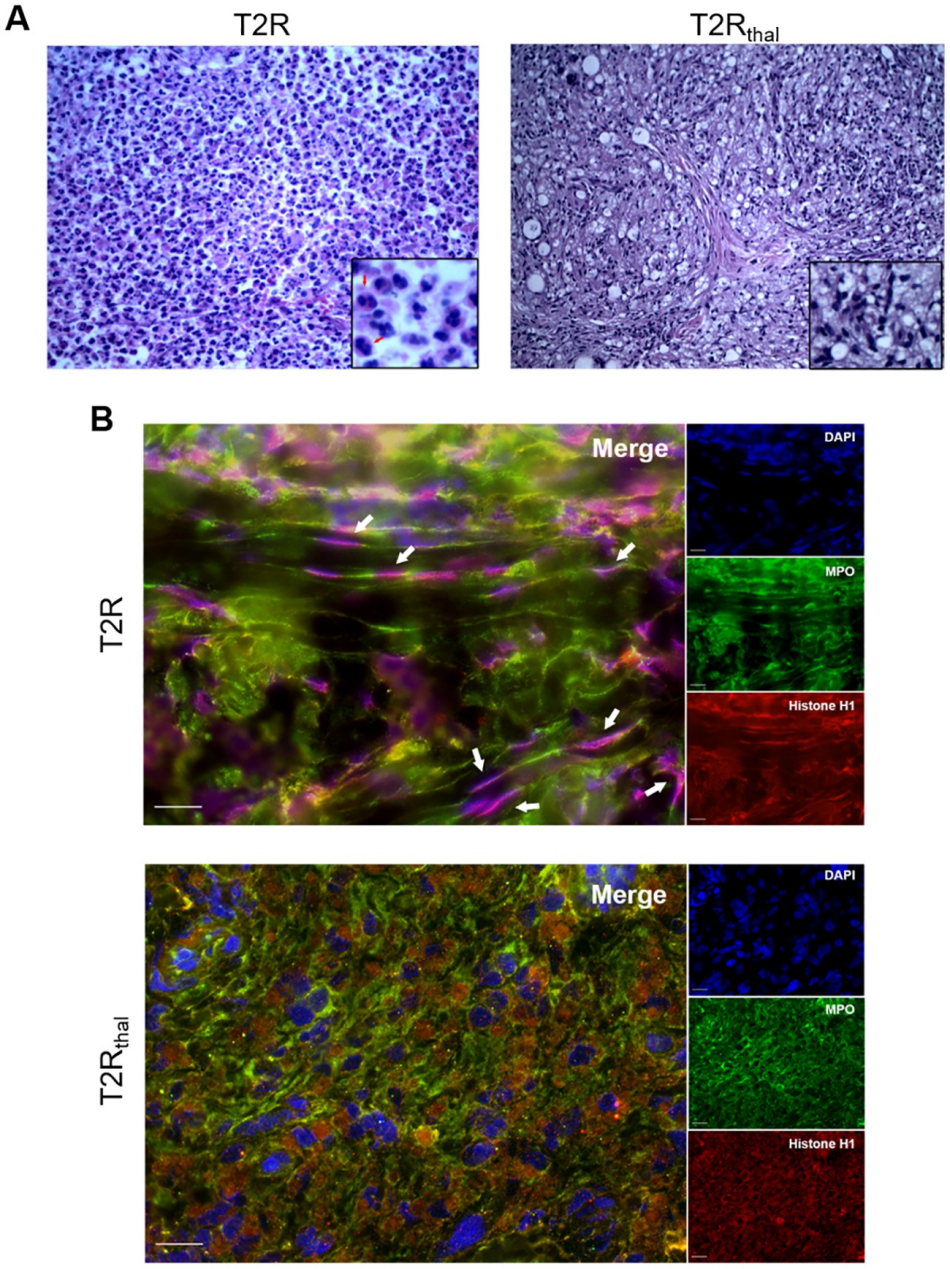
NETs are abundant in T2R skin lesions. (A) Histology of cutaneous lesions from T2R and T2R_thal_ patients stained by H&E. The arrows indicate the presence of neutrophils. Representative images of 3 patients (400x). (B) Skin lesions of T2R and T2R_thal_ patients were processed for immunostaining of NETs (MPO, green; histone H1, red; and DNA, blue). The arrows indicate the presence of NETs characterized by filamentous structures stained for these 3 macromolecules. The images are representative of 3 patients. Scale bar: 20 μm.

In most cases, the severe inflammatory process triggered during T2R leads to neutrophil infiltration [8]. Thus, as a first step, the presence of NETs in the skin lesions of T2R patients along with the potential effect of thalidomide on the abundance in NETs production were analyzed. Skin biopsy specimens stained with H&E confirmed the classical presence of an important inflammatory neutrophil infiltrate in the T2R lesion, which, importantly, was drastically reduced by day 7 of thalidomide treatment (T2R_Thal_) (Fig 1A) as previously described [9,20,21]. The lesions were then labeled for NETs detection by immunofluorescence, utilizing antibodies against MPO, a major component of neutrophil azurophilic granules, histone H1, and a DNA dye. Filamentous structures stained for these molecules characterizes NETs. It was observed intense NETs production in T2R patient skin lesions that essentially disappeared as a result of thalidomide treatment (Fig 1B).

**Fig 2:**
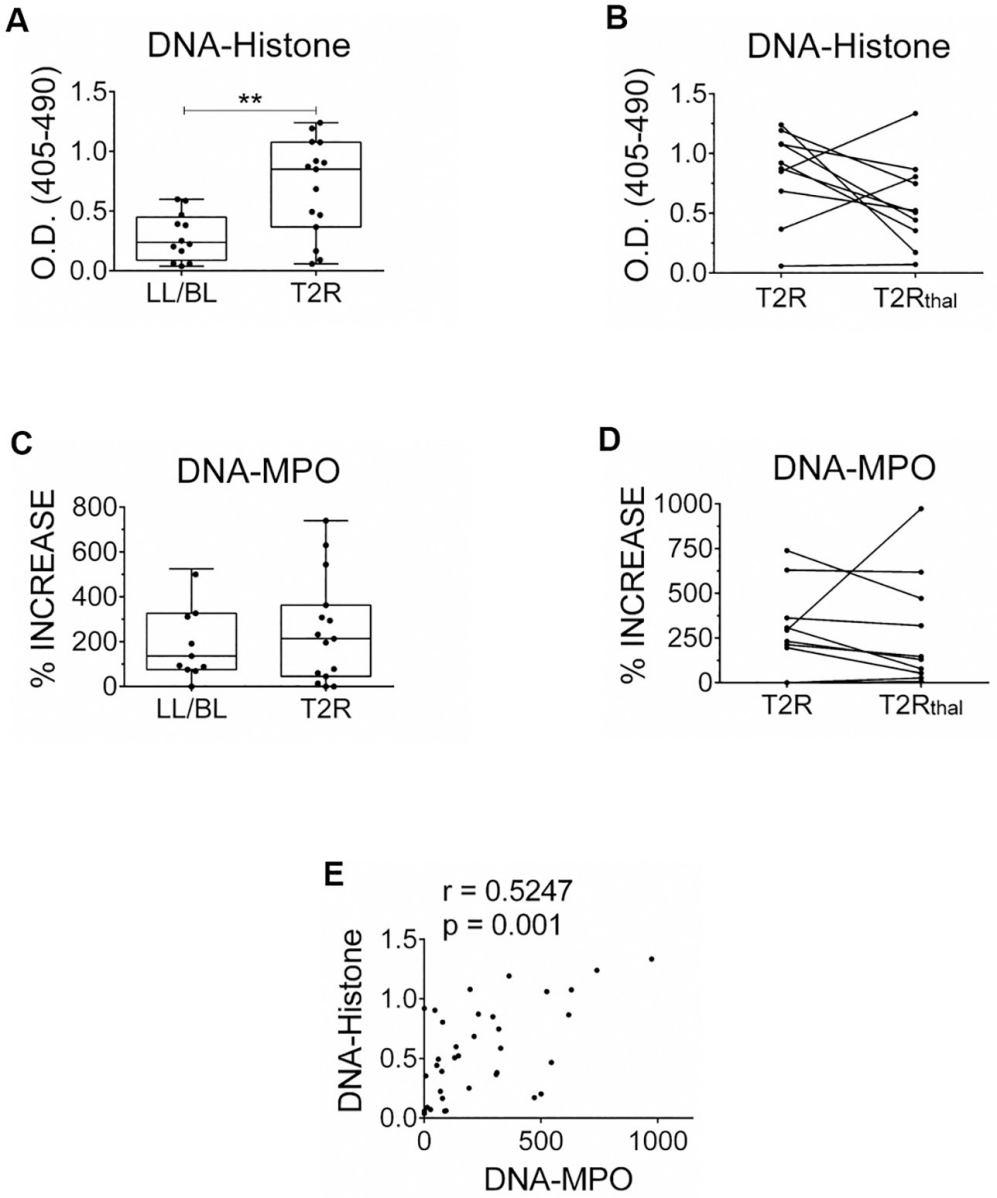
Circulating NETs components are higher in T2R patients, which decrease after thalidomide treatment. Patient serum samples were obtained; and the levels of the DNA-histone (A, B) and DNA-MPO (C, D) complexes were quantified by ELISA. DNA-MPO complex levels are expressed as percentages values above the blank. (B, D) Follow-up of the complexes serum levels during thalidomide treatment. Outliers were removed from analysis. Box plots show median, interquartile range, sample minimum, and maximum. Each dot represents a donor. **P<0.01 (Mann Whitney test). (E) Spearman’s correlation between DNA-histone and DNA-MPO complexes per patient (n = 36, r = 0.5247, P = 0.001).

Circulating NETs-derived components have been demonstrated in several inflammatory diseases in which NETosis has been implicated in their pathogenesis [22–24]. The levels of DNA-histone or DNA-MPO complexes in the serum of T2R patients were then investigated via specific immune enzymatic assays. In confirmation of our previous data [12], significantly higher human DNA-histone complex levels were found in T2R (median value of 0.8970 [minimum: 0.0580 –maximum: 1.240]) than in LL/BL patients (median value of 0.2380 [minimum: 0.0381 –maximum: 0.5995]) (Fig 2A). Moreover, a longitudinal follow-up of 10 T2R patients showed a reduction in DNA-histone complex levels in 7 of these patients at day 7 of treatment (T2R_thal_) (median value of 0.5156 [minimum: 0.0715 –maximum: 1.335]) (Fig 2B). The levels of this complex in healthy donors were similar to the ones in the LL/BL group, at a median value of 0.3645 (minimum: 0.1359 –maximum: 0.9568; n = 15). A trend toward higher levels of the serum DNA-MPO complex, a more specific marker of NETs, was observed in T2R patients (median value of 213,7% [minimum: 0%–maximum: 739.7%]) than those among non-reactional LL/BL (median value of 136.3% [minimum: 0%–maximum: 524.7%]) (Fig 2C). In addition, 7 out of the 10 T2R patients showed lower levels of this complex at day 7 of treatment (median value of 138.7% [minimum: 6.740%–maximum: 974.1%]) (Fig 2D). Healthy donors presented lower levels of this complex, at a median value of 6.780% (minimum: 0%–maximum: 54.64%; n = 15) over the blank. Notably, a positive correlation was found between DNA-histone and DNA-MPO complexes (r = 0.5247, P = 0.001; Fig 2E), reinforcing the idea that NETosis is an important source of the DNA-histone complexes detected in serum of T2R patients.

**Fig 3:**
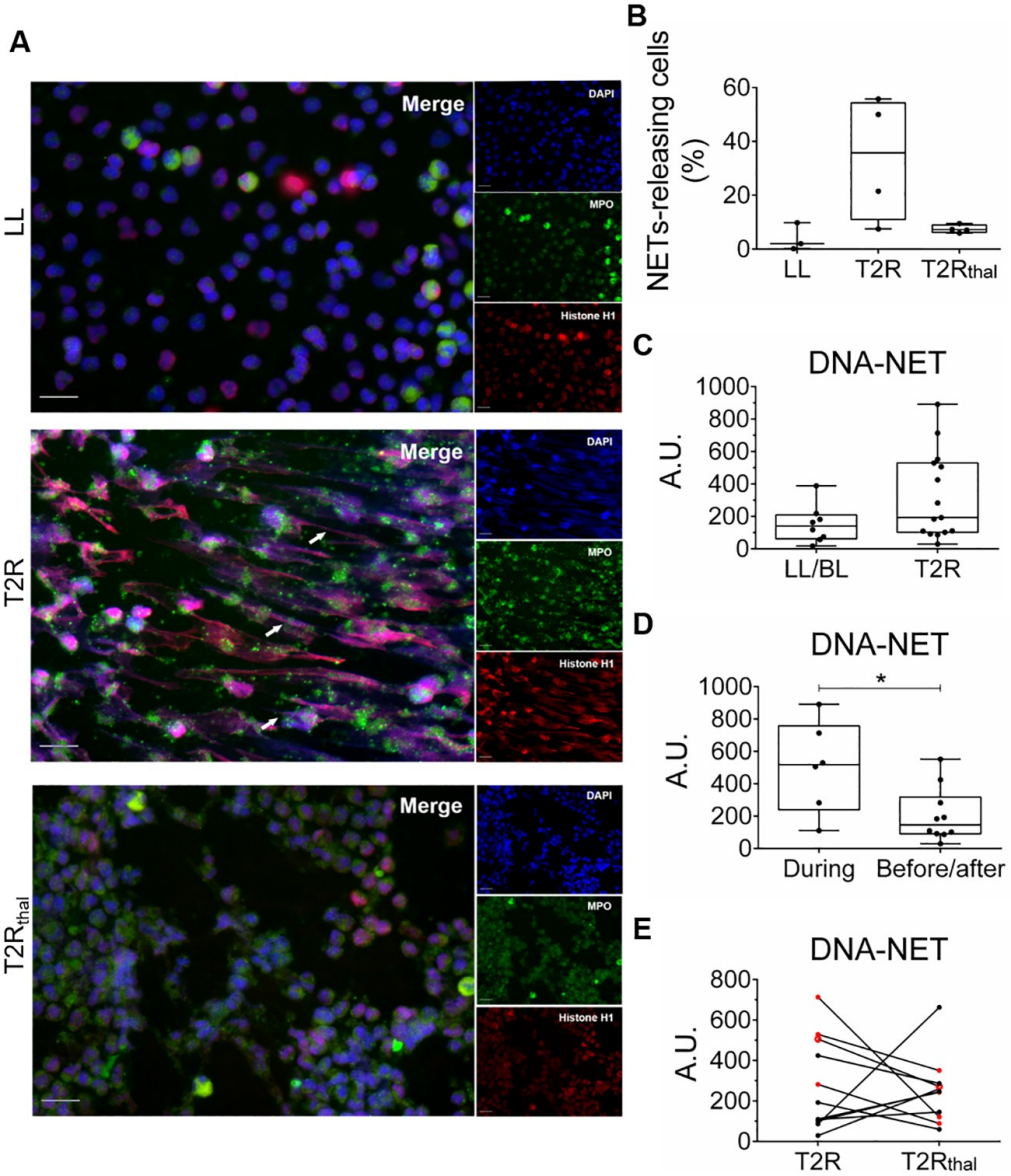
T2R neutrophils show spontaneous *ex vivo* NETs production, which decreases with thalidomide treatment. Neutrophils from LL/BL, T2R, and T2R_thal_ patients were isolated and incubated in medium alone for 90 min. (A) Immunostaining of NETs components (MPO, green; histone, red; and DNA, blue). Arrows indicate the presence of NETs. Images are representative of 3 LL/BL, 4 T2R and 4 T2R_thal_. Scale bar: 20 μm. (B) The percentage of NETs-releasing cells from 5 fields captured from 3 LL/BL, 4 T2R and 4 T2R_thal_ patients is shown. (C) Neutrophils from LL/BL, T2R, and T2R_thal_ patients were incubated for 90 min and DNA release was measured by picogreen. Box plots show median, interquartile range, sample minimum, and maximum. Each dot represents a donor. (D) Comparison of patients who developed T2R during MDT versus those with reactional episodes before or after MDT. (E) Follow-up of DNA levels released after 7 days of thalidomide treatment. Dots in red are patients who developed T2R during MDT. *P<0.05 (Mann Whitney test).

Peripheral neutrophils undergoing NETosis have been described in SLE, rheumatoid arthritis, and psoriasis [24–26]. So, as a next step, the production of NETs by T2R peripheral neutrophils in the absence of any stimulus was investigated. Blood was obtained, neutrophils were isolated, and their capacity to undergo *ex vivo* spontaneous NETosis was evaluated by fluorescence microscopy after a 90-min incubation period. Neutrophil preparations showed an average > 98% purity, as determined via microscopic analysis and flow cytometry (S1 Fig). Immunofluorescence staining of NETs clearly showed spontaneous NETs formation by neutrophils isolated from T2R patients when compared to neutrophils isolated from non-reactional LL/BL and T2R_thal_ patient (Fig 3A). The percentage of NETs-releasing cells was quantified in images from 3 LL/BL, 4 T2R and 4 T2R_thal_. Despite the high differences in the rates of NETs formation observed among T2R patients, neutrophils from these patients showed higher *ex vivo* spontaneous NETs production than non-reactional LL/BL patients with a subsequent drastic decrease after thalidomide treatment (Fig 3B). Spontaneous *ex vivo* NETs production was then evaluated in a larger number of patients by quantifying DNA release in culture supernatants. In agreement with the immunofluorescence results, variable levels of released DNA were detected among LL/BL and T2R patients with a trend of higher levels in reactional patients (Fig 3C). Interestingly, among the 7 T2R patients with the highest DNA values, 5 developed reaction during MDT. Indeed, when this group was compared with the T2R patients who developed reaction before or after MDT, a significant difference was observed between them (Fig 3D). All T2R patients with the highest DNA levels showed reduced DNA release after 7 days of thalidomide medication (Fig 3E). Altogether, these data indicate that excessive NETs formation occurs locally in the skin lesions and circulation of T2R patients and that this phenomenon tends to decline in thalidomide-treated patients.

The higher levels of spontaneous NETs formation observed in reactional MDT-T2R patients (Fig 3D) may be related to the massive release of ML components from infected tissue taking place during treatment. Since bacterial components are known to trigger NETosis [27], the capacity of the ML whole-cell sonicate (MLWCS) to induce NETs formation *in vitro* was investigated. S2A Fig outlines the dose-response curve of DNA release by neutrophils isolated from healthy donors stimulated for 90 min with MLWCS. The concentration of 20 μg/mL induced significantly higher levels of NETs production relative to those in non-stimulated cells and, as a result, was used in the remaining assays of the study. Time-course assays were also performed, indicating 90 min as the best time point for NETs formation in response to MLWCS (S2B Fig). The activity of LDH was measured in culture supernatants indicating that cells were not dying of necrosis (S2C Fig). S3 Fig shows the immunofluorescence image of MLWCS-induced NETs, confirming the capacity of MLWCS to induce NETs formation in healthy donor neutrophils *in vitro*. It can be clearly seen that MLWCS-induced NETs are similar to those observed in neutrophils stimulated with PMA, a classical NETosis inducer (S3 Fig).

**Fig 4:**
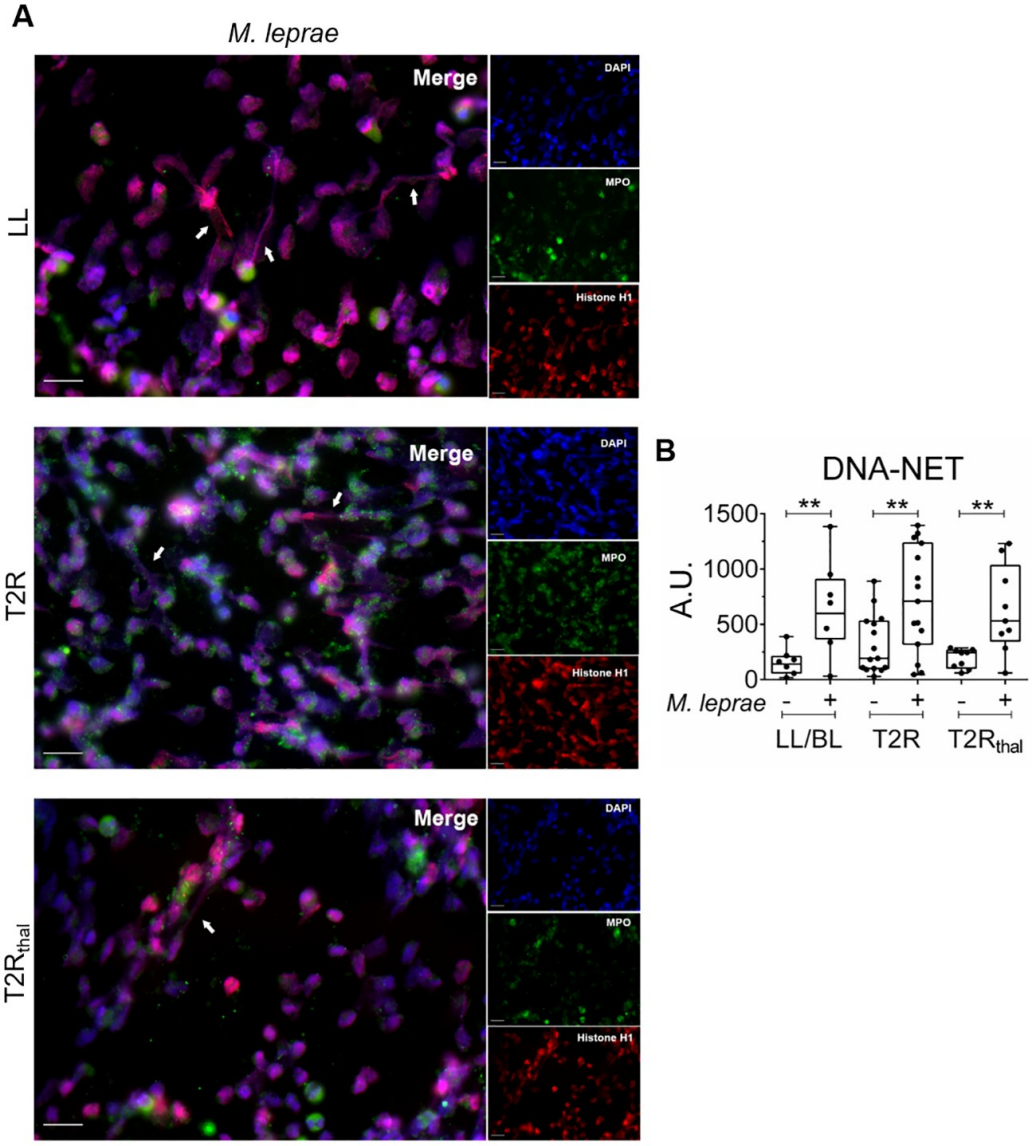
*M*. *leprae* induces NETs formation *in vitro*. Neutrophils from LL/BL, T2R, and T2R_thal_ patients were isolated and stimulated or not with *M*. *leprae* whole-cell sonicate (MLWCS) for 90 min. (A) Immunostaining of NETs components (MPO, green; histone, red; and DNA, blue). Arrows indicate the presence of NETs. Images are representative of 3 LL/BL, 4 T2R, and 4 T2R_thal_. Scale bar: 20 μm. (B) DNA release was measured in culture supernatants by picogreen. Box plots show median, interquartile range, sample minimum, and maximum. Each dot represents a donor. **P<0.01 (Mann Whitney test).

Next, the ability of MLWCS to induce NETs production in leprosy patient peripheral neutrophils was examined. NETs formation was clearly demonstrated by immunofluorescence (Fig 4A). The image of the T2R is from a patient with low spontaneus NETs production, so that the potential capacity of MLWCS to induce NETs formation could be more easily visualized. When NET-DNA was quantified in culture supernatants, it was clearly observed that MLWCS is able to stimulate the neutrophils of all 3 groups of patients to produce NETs at higher levels than in the non-stimulated cultures (Fig 4B). Interesting, the BI values of T2R patients (at the time of leprosy diagnosis) and the serum DNA-histone levels showed a positive correlation (r = 0.52 and P = 0.046), reinforcing the idea that bacterial components trigger NETosis during TLR (S4 Fig).

The apparently *in vivo* inhibitory effect of thalidomide on NETs production in T2R patients observed both at the lesion site (Fig 1B) and in peripheral neutrophils (Fig 3) raised the hypothesis that the drug might be acting directly in this process. As a test, healthy-donor neutrophils were stimulated *in vitro* with MLWCS in the presence or not of thalidomide while NETs production was monitored by DNA release. S5A Fig shows the results obtained with one donor, in which no effect of thalidomide on MLWCS-induced NET formation was observed. S5B Fig summarizes the results of 6 healthy donors, only one of whom experienced an inhibitory effect. As expected, however, thalidomide was able to block TNF secretion by monocytes in response to LPS, as previously referred to [17] (S5C Fig). The incapacity of thalidomide to directly interfere in the NETs production process is reinforced by the observation that T2R_thal_ neutrophils continued to respond to MLCWS *in vitro* even after receiving *in vivo* thalidomide (Fig 4B). These data suggest that the inhibitory *in vivo* effect of thalidomide on NETs formation is, albeit indirectly. One possible mechanism could be related to the capacity of this drug to block the production of inflammatory mediators. NETs are generated in an inflammatory milieu and several pro-inflammatory cytokines have been shown to induce NETosis. The capacity of thalidomide to decrease the levels of pro-inflammatory cytokines during T2R treatment both locally at the skin lesions as well as systemically has been well demonstrated [20,28]. Particularly TNF, which has been proposed to play a major role in T2R pathogenesis, shows a drastic reduction even few days after the onset of thalidomide treatment, and has been shown to induce NETosis *in vitro* [29]. Another candidate could be IFN-I, preferentially present in skin lesions of lepromatous leprosy patients [30], and known to prime neutrophils to NETosis [31] and to be inhibited by thalidomide [32]. It will be relevant to analyze in future studies whether T2R patients treated with steroids show comparable inhibitory effects on NETs formation as thalidomide treated patients. Due to thalidomide restricted use, most countries use steroids for the managements of T2R patients, although these drugs are less effective in remission of T2R symptoms [33]. Interestingly, a study by Lapponi *et al*. (2013) observed no inhibitory effect of dexamethasone on NET formation, both *in vitro* in isolated human neutrophils, as well as *in vivo* using a mouse model of peritonitis [29].

**Fig 5:**
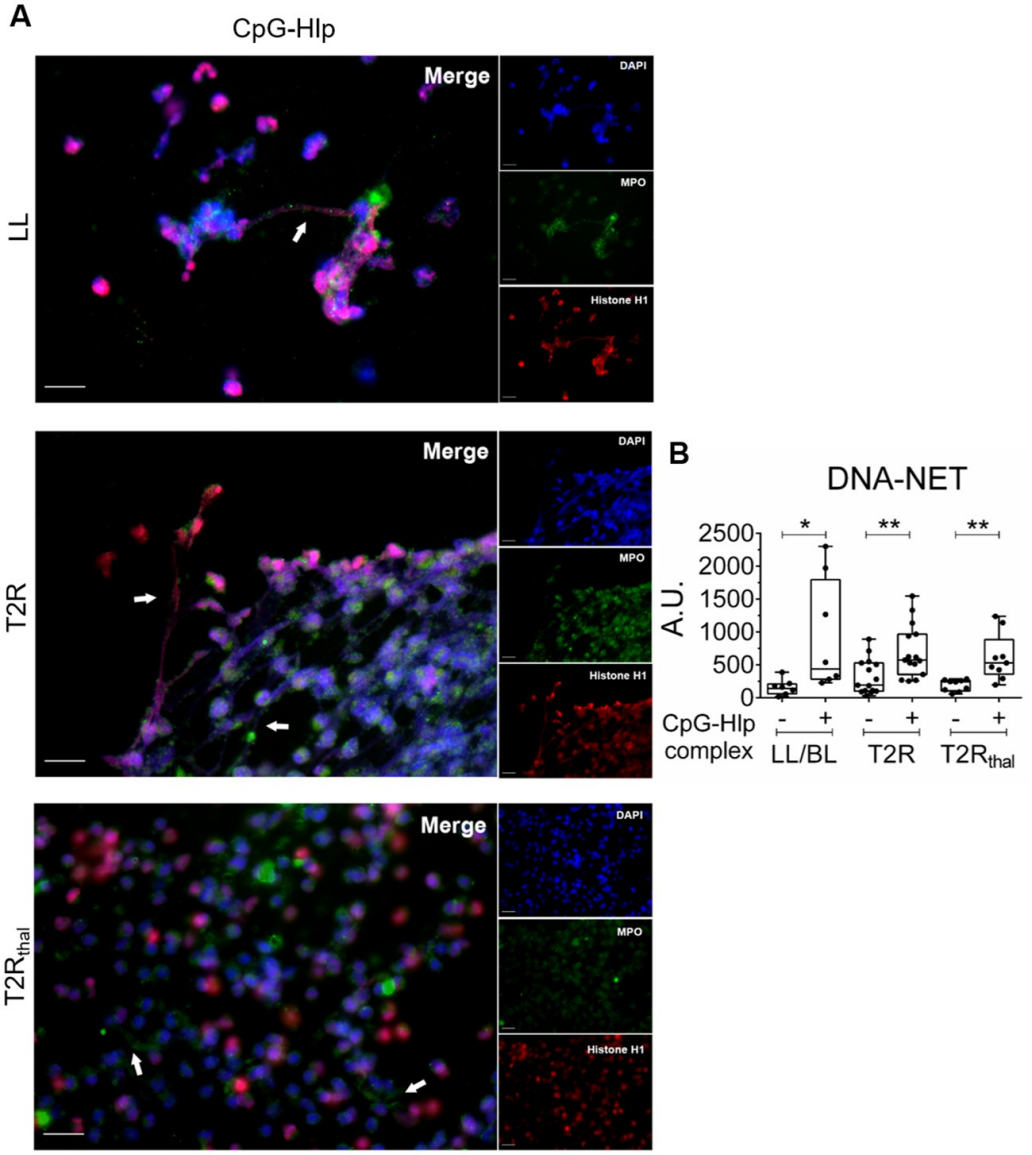
CpG-Hlp, a mycobacterial TLR9 ligand, induces NETs formation *in vitro*. Neutrophils from LL/BL, T2R, and T2R_thal_ patients were isolated and stimulated or not with CpG-Hlp complex for 90 min. (A) Immunostaining of NETs components (MPO, green; histone, red; and DNA, blue). Arrows indicate the presence of NETs. Images are representative of 3 LL/BL, 4 T2R, and 4 T2R_thal_. Scale bar: 20 μm. (B) DNA release was measured in culture supernatants with picogreen. Box plots show median, interquartile range, sample minimum, and maximum. Each dot represents a donor. *P<0.05 and **P<0.01 (Mann Whitney test).

TLRs had been previously implicated in NETosis induction [27]. In our previous study, besides the higher levels of endogenous DNA-histone complexes, higher levels of the mycobacterial histone-like protein (Hlp) in T2R patients sera, probably released as a consequence of bacterial killing during MDT [12], were also detected. Thus, we hypothesized that Hlp complexed to DNA, a TLR9 ligand [12,34], could be one of the bacterial components present in MLWCS that is responsible for NETs induction. To test this hypothesis, recombinant Hlp was obtained and then combined with the CpG oligonucleotide, mimicking bacterial DNA. Neutrophils were then stimulated with the CpG-Hlp complex for 90 min. The concentration of the CpG-Hlp complex used in the assays was based on the dose-response curves obtained from 2 healthy-donor neutrophils (S6A Fig). At this concentration, Hlp alone was unable to induce DNA release, but when combined to CpG was able to enhance the effects induced by CpG alone (S6B Fig), as shown in previous studies [12,34]. The capacity of CpG-Hlp to stimulate NETs formation in healthy donor and leprosy patients neutrophils was demonstrated by immunofluorescence (S3 Fig and Fig 5A, respectively). NETs production was quantified by measuring DNA release in culture supernatants. CpG-Hlp was shown to induce NET formation *in vitro* in leprosy patient neutrophils at levels comparable to those seen for MLWCS (Fig 5B).

**Fig 6:**
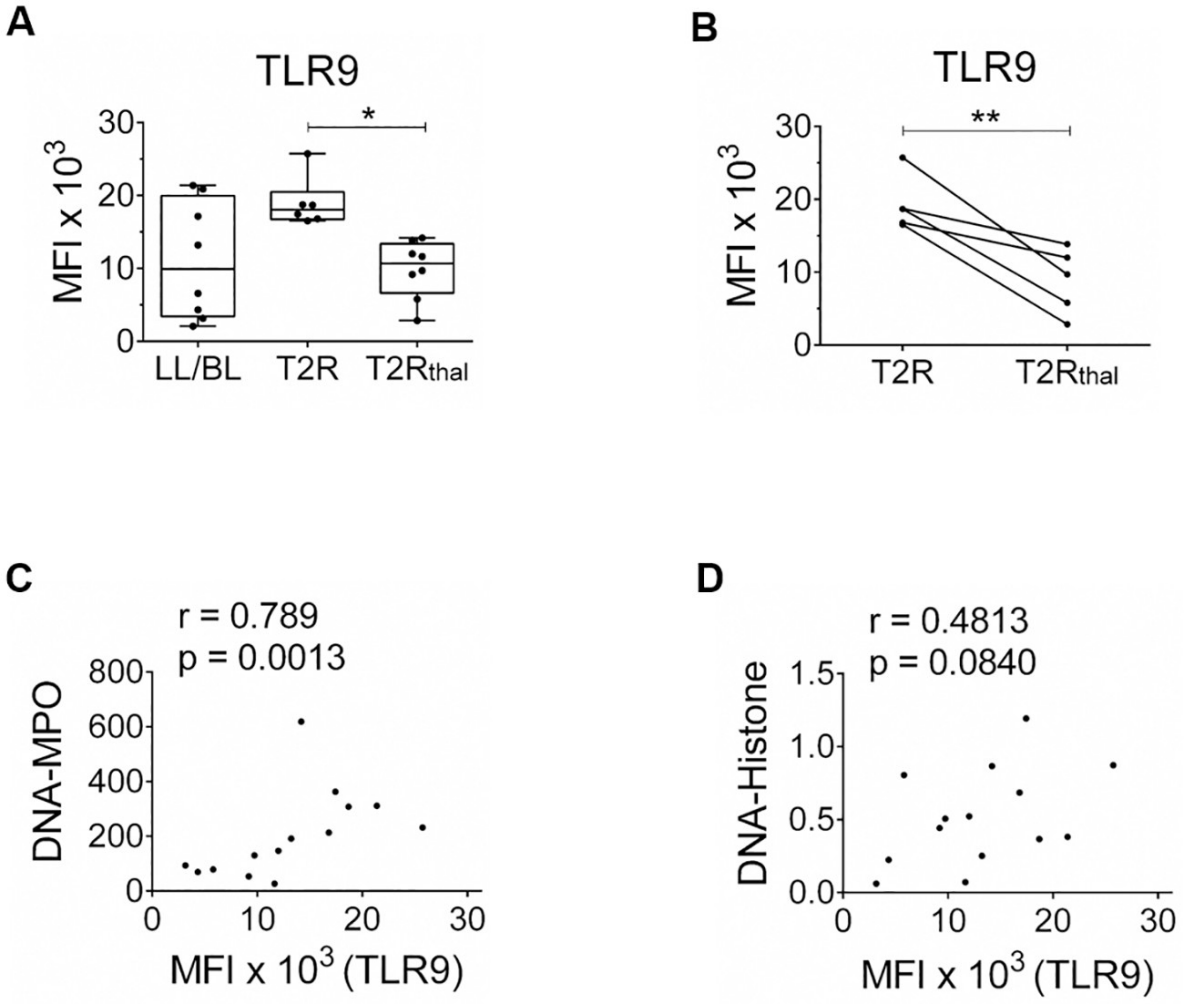
T2R patient neutrophils express higher levels of TLR9, which decrease after thalidomide treatment. (A) Neutrophils of LL/BL, T2R and T2R_thal_ patients were isolated and *ex vivo* TLR9 expression levels were quantified by flow cytometry. *P<0.05 (Kruskal-Wallis test). (B) Follow-up of TLR9 expression in T2R patients after 7 days of thalidomide treatment. Box plots show median, interquartile range, sample minimum, and maximum. Each dot represents a donor. **P<0.01 (Wilcoxon test). (C) Spearman’s correlation between DNA-MPO complex and TLR9 expression per patient (n = 14, r = 0.789, P = 0.0013). (D) Spearman’s correlation between DNA-histone complex and TLR9 expression per patient (n = 14, r = 0.4813, P = 0.0840).

We have previously demonstrated higher expression levels of TLR9 in skin lesions and T2R blood mononuclear cells as opposed to LL/BL patient levels [12]. Thus, as a next step, the status of TLR9 expression in leprosy patient neutrophils was compared. The median fluorescence intensity (MFI) values of TLR9 in T2R neutrophils were higher overall relative to those of LL/BL patients. Moreover, a significant decrease in TLR9 expression was observed in T2R_thal_, reaching values close to the ones seen in LL/BL patients (Fig 6A). Indeed, during follow-up of 5 T2R patients, an accentuated drop in TLR9 expression after 7 days of thalidomide treatment was shown (Fig 6B). Representative histograms are exhibited in S7 Fig. It is noteworthy that although T2R and T2R_thal_ neutrophils express different TLR9 levels, both demonstrated a similar capacity to respond *in vitro* to CpG-Hlp. One possible explanation for this observation may be the high concentration of CpG-Hlp used in these *in vitro* assays, constituting a supra-optimal stimulation (S6A Fig). However, the involvement of other nucleic acid sensors cannot be ruled out. Moreover, T2R_thal_ neutrophils also preserved the capacity to responde to MLWCS (Fig 4B), which may suggest that other innate recognition pathways besides TLR9 might be enrolled in NETosis induction when *M*. *leprae* whole cell extract is used as stimulus. Of note, a positive correlation was observed between the levels of TLR9 expression in neutrophils and circulating amounts of DNA-MPO, but not of DNA-Histone, in leprosy patients (Fig 6C and 6D).

The capacity of CpG-Hlp to induce NETs formation *in vitro* together with the higher levels of TLR9 expression and the simultaneous increase in the circulating levels of its ligands (both the human DNA-histone and -Hlp complexes [12]) strongly suggest that DNA recognition via TLR9 is involved in NETs production during T2R. Both human DNA-histone complexes and TLR9 levels decreased after 7 days of treatment with thalidomide (Figs 2 and 6), concomitant with a diminished spontaneous NETs formation (Fig 3). It is worth mentioning again that thalidomide is an extremely effective drug in the remission of T2R clinical symptoms [35,36]. Thus, the blockage of NETs formation observed in treated patients reinforces the idea that excessive NETs generation may play an important role in T2R pathogenesis.

In summary, our study sheds lights on the pathogenesis of T2R, pointing to NETosis as an important source of extracellular DNA and autoantigens, known to activate a plethora of immunoinflammatory pathways. This conclusion is based on the following findings: i) abundant NETs were found in T2R skin lesions; ii) higher serum levels of potential NETs markers were detected in T2R patients; iii) increased spontaneous NETs formation was observed in T2R peripheral neutrophils; iv) both *M*. *leprae* whole-cell sonicate and CpG-Hlp complex, mimicking a mycobacterial TLR9 ligand, were able to induce NETs production *in vitro*; and finally v) administration of thalidomide for 7 consecutive days to T2R patients, a very effective drug for T2R treatment, resulted in a decrease in all NETosis parameters evaluated *in vivo* and *ex vivo*.

NETs have been implicated in the immunopathogenesis of SLE and other chronic inflammatory autoimmune diseases via the following mechanisms: 1) the externalization of modified autoantigens; 2) induction of type I IFN; 3) autoantibody production; 4) stimulation of the inflammasome; 5) activation of the classical and alternative pathways of the complement system; and 6) direct effects on the endothelium and the consequent induction of vasculopathy [37]. Since most of these events have also been shown to be activated during T2R [7], it is reasonable to speculate that the excessive NETs production herein described performs a central role in triggering and amplifying these pathological pathways in T2R.

In terms of practical implications for the management of T2R patients, the data here generated may contribute to the identification of biomarkers for early diagnosis and for the emergence of novel alternative therapies in the near future. Indeed, the potential prognostic values for increased serum levels of NETs markers should be explored in future prospective follow-up studies of multibacillary leprosy patients. Moreover, novel therapies under development to treat autoimmune inflammatory diseases that target the accumulation of NETs [38] may prove useful in treating T2R.

## Supporting information

S1 Fig Rate of purity of isolated neutrophils. (A-C) Representative dot-plot diagram of the flow cytometry of neutrophils isolated from healthy donors for rate-of-purity evaluation. (A) *Gate* generated by FSC-A vs. FSC-H parameters for singlet analysis. (B) The granulocytic population *gate* was generated by FSC-A vs. SSC-A parameters. (C) The percentage of CD16^+^ cells was provided by gating FSC-A *vs*. CD16 axes. Representative of 4 healthy donors. (D) Representative image of a cytospin slide of purified neutrophils (n = 4; 100x).
(TIF)

S2 Fig *M*. *leprae* induces NET formation. (A) Healthy-donor neutrophils (1x10^6^; n = 6) were stimulated or not with *M*. *leprae* whole-cell sonicate (MLWCS) at different concentrations (1, 5, 10, and 20 μg/mL) for 90-min incubation. DNA release in the supernatant was measured by picogreen. Box plots show median, interquartile range, sample minimum, and maximum. Each dot represents a donor. ***P<0.001 (Kruskal-Wallis test). (B, C) Neutrophils (1x10^6^) were stimulated or not with 20 μg/mL of MLWCS for 60, 90, 120, and 180 min. DNA release was measured by picogreen (B) and lactate dehydrogenase (LDH) enzyme activity was determinated using the Liquiform LDH kit (C). Dimethylsulfoxide (DMSO; 20%) was included as a positive control for necrosis induction. Representative of 3 individuals.
(TIF)

S3 Fig *M*. *leprae* and CpG-Hlp complex induce NET formation *in vitro*. Healthy-donor Neutrophils (2x10^6^ cells) were stimulated or not with MLWCS (20 μg/mL), CpG-Hlp complex (0.5 μM-0.25 μM) and 200 ng/mL PMA (positive control) for 90-min incubation. Immunostaining of NETs components (MPO, green; histone, red; and DNA, blue). Arrows indicate the presence of NETs. Representative immunofluorescence images of 4 individuals. Scale bar: 20 μm.
(TIF)

S4 Fig Association between Bacteriological index (BI) and serum levels of DNA-histone and -MPO complexes in T2R patients. (A) Spearman’s correlation between BI and DNA-histone complex (n = 15, r = 0.5254, P = 0.0466). (B) Spearman’s correlation between BI and DNA-MPO complex (n = 15, r = 0.4559, P = 0.0892).
(TIF)

S5 Fig *In vit*ro effect of thalidomide on *M*. *leprae*-induced NETs formation. (A) Neutrophils (1x10^6^ cells) from healthy donors were stimulated or not with MLWCS (20 μg/mL) and/or thalidomide (50 μg/mL) for 90-min incubation; and DNA release was measured by picogreen. Representative of 6 healthy donors. (B) DNA release by healthy-donor neutrophils (n = 6) stimulated with MLWCS in the presence or absence of thalidomide. Each dot represents a donor. (C) To test the efficacy of *in vitro* thalidomide, monocytes (2x10^6^ cells) from healthy donors were stimulated or not with LPS (1 μg/mL) and/or thalidomide (50 μg/mL) for an 18h-incubation period for TNF release dosing by ELISA. Data represent median of 2 healthy donors. DMSO was used as vehicle.
(TIF)

S6 Fig Induction of NETs release by TLR9 ligand. (A) Neutrophils from healthy donors were stimulated with different concentrations of CpG-Hlp complex for 90 min and DNA release was measured by picogreen. (B) Healthy neutrophils were stimulated with CpG (0.5 μM), Hlp (0.25 μM), or CpG-Hlp (0.5 μM-0.25 μM) for 90-min incubation and DNA release was measured by picogreen. Box plots show median, interquartile range, sample minimum, and maximum. Each dot represents a donor.
(TIF)

S7 Fig Levels of TLR9 expression in leprosy patient neutrophils. Representative histograms showing the quality of anti-TLR9 antibody labeling in neutrophils isolated from the different groups of analyzed patients.
(TIF)
