# Supplementary figures and images for: Neutrophil extracellular traps contribute to the pathogenesis of leprosy type 2 reactions
Source: PLoS Negl Trop Dis. 2019 Sep 10;13(9):e0007368. doi: 10.1371/journal.pntd.0007368 (PMC6736252; doi:10.1371/journal.pntd.0007368)

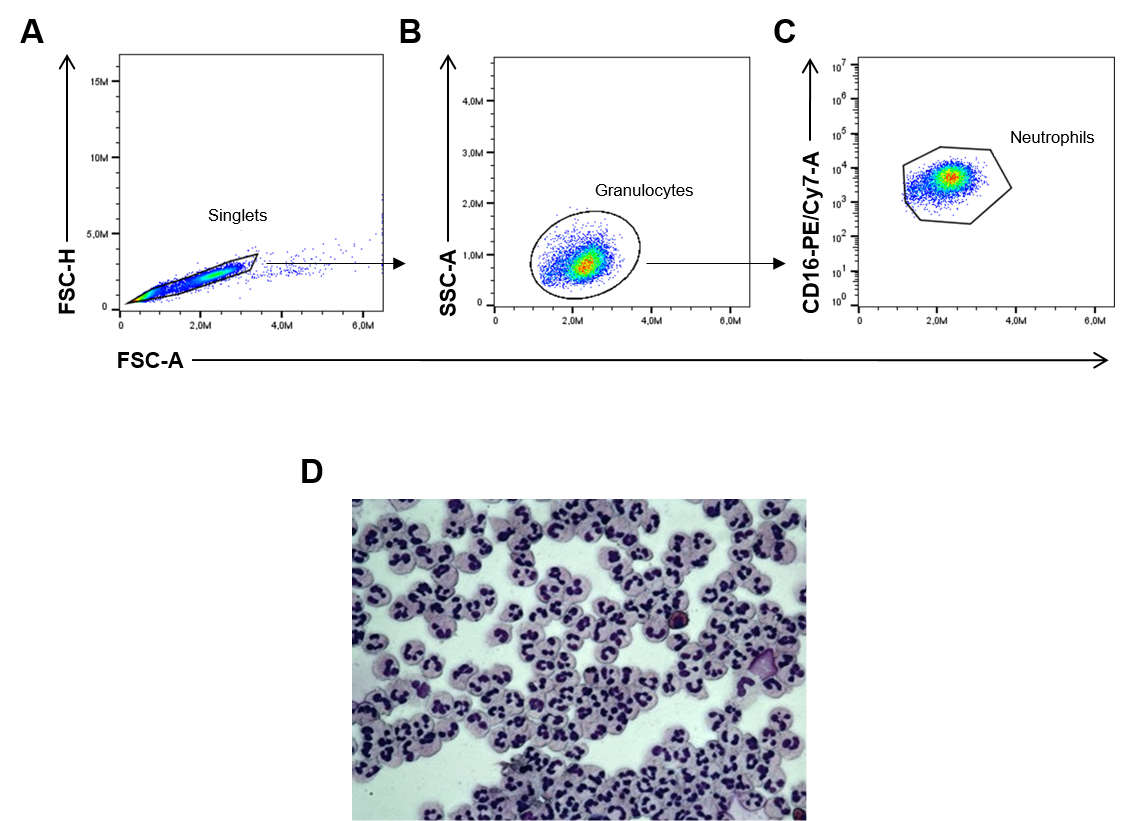

Supplement: S1 Fig — (A-C) Representative dot-plot diagram of the flow cytometry of neutrophils isolated from healthy donors for rate-of-purity evaluation. (A) Gate generated by FSC-A vs. FSC-H parameters for singlet analysis. (B) The granulocytic population gate was generated by FSC-A vs. SSC-A parameters. (C) The percentage of CD16+ cells was provided by gating FSC-A vs. CD16 axes. Representative of 4 healthy donors. (D) Representative image of a cytospin slide of purified neutrophils (n = 4; 100x). (TIF) [file pntd.0007368.s001.tif]

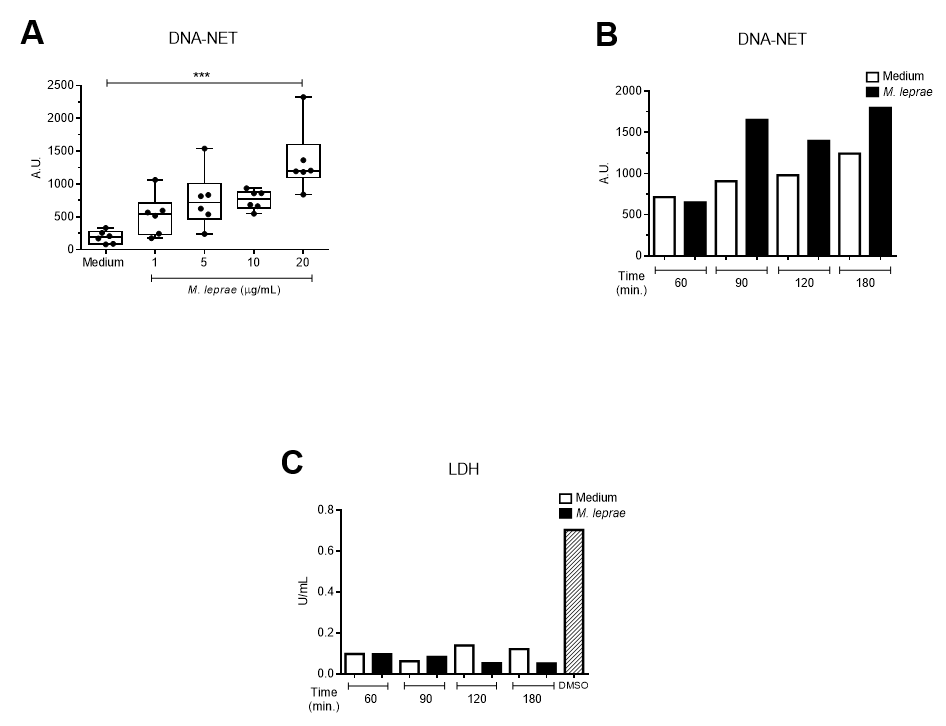

Supplement: S2 Fig — (A) Healthy-donor neutrophils (1x10^6^; n = 6) were stimulated or not with M. leprae whole-cell sonicate (MLWCS) at different concentrations (1, 5, 10, and 20 μg/mL) for 90-min incubation. DNA release in the supernatant was measured by picogreen. Box plots show median, interquartile range, sample minimum, and maximum. Each dot represents a donor. ***P<0.001 (Kruskal-Wallis test). (B, C) Neutrophils (1x10^6^) were stimulated or not with 20 μg/mL of MLWCS for 60, 90, 120, and 180 min. DNA release was measured by picogreen (B) and lactate dehydrogenase (LDH) enzyme activity was determinated using the Liquiform LDH kit (C). Dimethylsulfoxide (DMSO; 20%) was included as a positive control for necrosis induction. Representative of 3 individuals. (TIF) [file pntd.0007368.s002.tif]

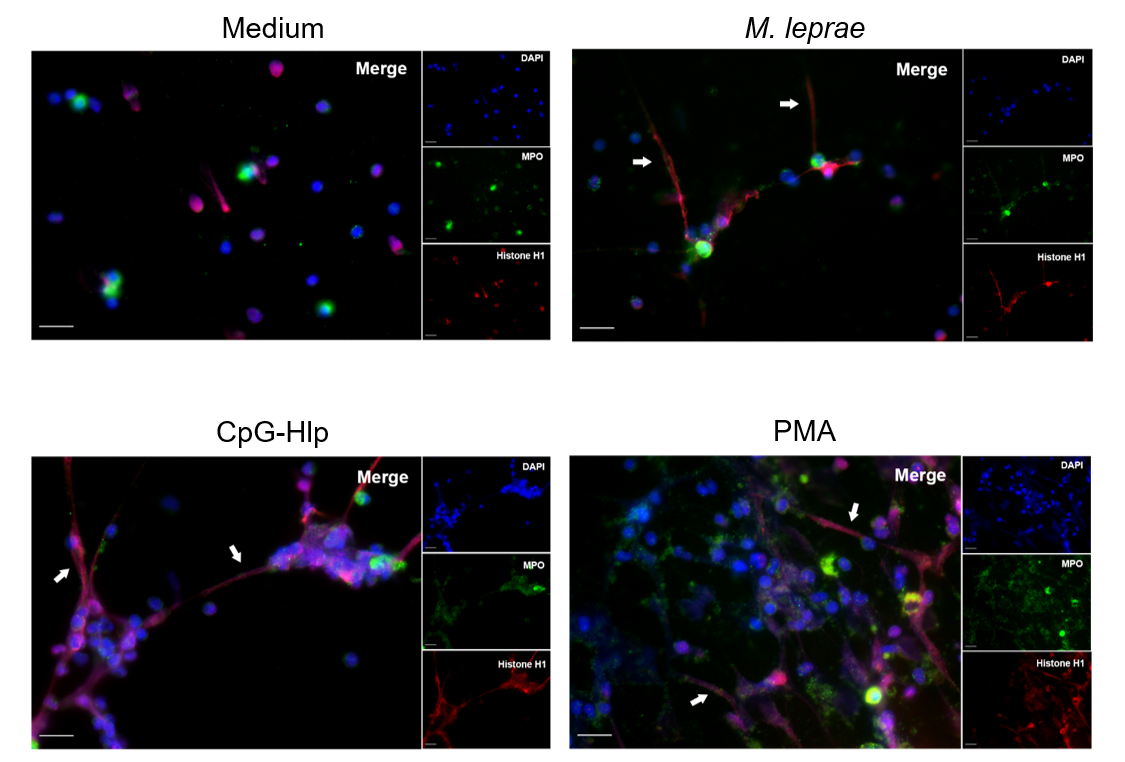

Supplement: S3 Fig — Healthy-donor Neutrophils (2x10^6^ cells) were stimulated or not with MLWCS (20 μg/mL), CpG-Hlp complex (0.5 μM-0.25 μM) and 200 ng/mL PMA (positive control) for 90-min incubation. Immunostaining of NETs components (MPO, green; histone, red; and DNA, blue). Arrows indicate the presence of NETs. Representative immunofluorescence images of 4 individuals. Scale bar: 20 μm. (TIF) [file pntd.0007368.s003.tif]

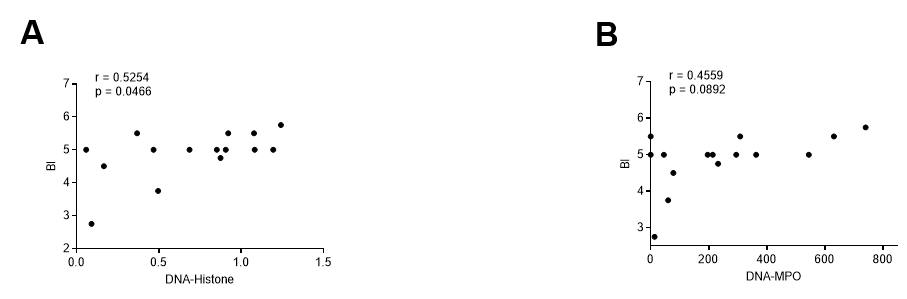

Supplement: S4 Fig — (A) Spearman’s correlation between BI and DNA-histone complex (n = 15, r = 0.5254, P = 0.0466). (B) Spearman’s correlation between BI and DNA-MPO complex (n = 15, r = 0.4559, P = 0.0892). (TIF) [file pntd.0007368.s004.tif]

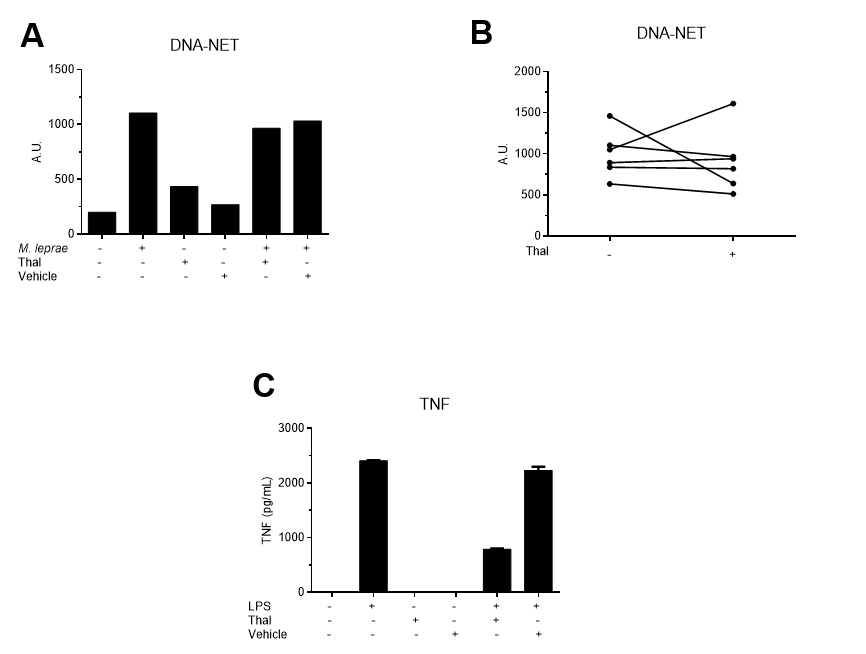

Supplement: S5 Fig — (A) Neutrophils (1x10^6^ cells) from healthy donors were stimulated or not with MLWCS (20 μg/mL) and/or thalidomide (50 μg/mL) for 90-min incubation; and DNA release was measured by picogreen. Representative of 6 healthy donors. (B) DNA release by healthy-donor neutrophils (n = 6) stimulated with MLWCS in the presence or absence of thalidomide. Each dot represents a donor. (C) To test the efficacy of in vitro thalidomide, monocytes (2x10^6^ cells) from healthy donors were stimulated or not with LPS (1 μg/mL) and/or thalidomide (50 μg/mL) for an 18h-incubation period for TNF release dosing by ELISA. Data represent median of 2 healthy donors. DMSO was used as vehicle. (TIF) [file pntd.0007368.s005.tif]

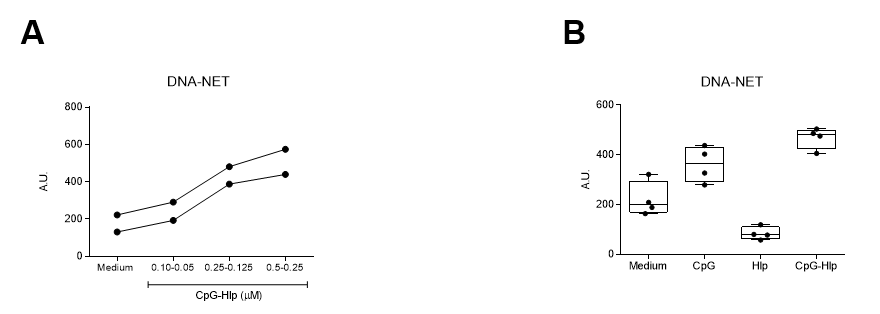

Supplement: S6 Fig — (A) Neutrophils from healthy donors were stimulated with different concentrations of CpG-Hlp complex for 90 min and DNA release was measured by picogreen. (B) Healthy neutrophils were stimulated with CpG (0.5 μM), Hlp (0.25 μM), or CpG-Hlp (0.5 μM-0.25 μM) for 90-min incubation and DNA release was measured by picogreen. Box plots show median, interquartile range, sample minimum, and maximum. Each dot represents a donor. (TIF) [file pntd.0007368.s006.tif]

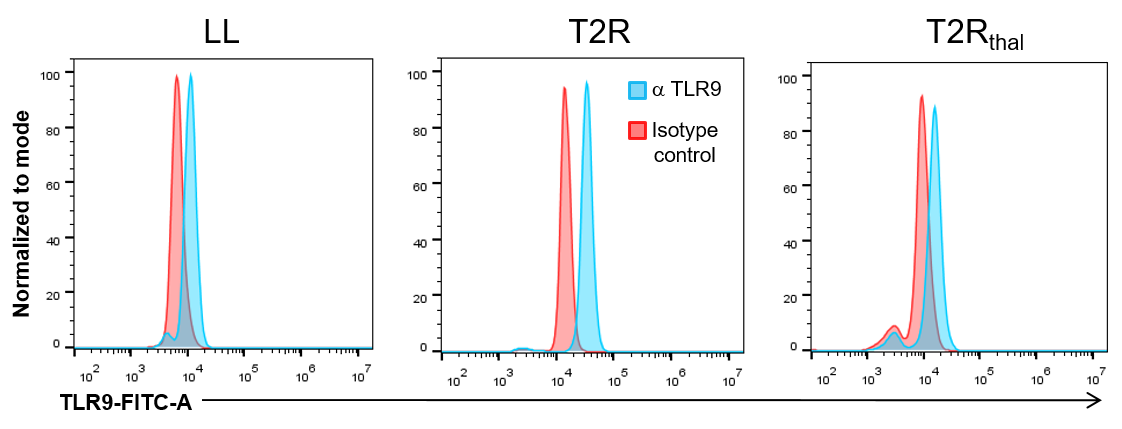

Supplement: S7 Fig — Representative histograms showing the quality of anti-TLR9 antibody labeling in neutrophils isolated from the different groups of analyzed patients. (TIF) [file pntd.0007368.s007.tif]
